# Safety and feasibility of tracheostomy and gastrostomy home replacement: a five-year experience from a palliative care center

**DOI:** 10.3389/fped.2025.1644830

**Published:** 2025-09-04

**Authors:** Francesca Burlo, Irene Bortolin, Domenico Leonardo Grasso, Anna Oveglia, Egidio Barbi, Lucia De Zen, Valentina Taucar, Francesca Peri

**Affiliations:** ^1^ Department of Medicine, Surgery, and Health Sciences, University of Trieste, Trieste, Italy; ^2^ Unit of Pediatric Audiology and Otorhinolaryngology, Institute of Maternal and Child Health—IRCCS Burlo Garofolo, Trieste, Italy; ^3^ Pediatric Palliative Care and Pain Service, Institute for Maternal and Child Health—IRCCS Burlo Garofolo, Trieste, Italy; ^4^ Department of Pediatric, Institute for Maternal and Child Health—IRCCS Burlo Garofolo, Trieste, Italy

**Keywords:** palliative care, home assistance, tracheostomy, gastrostomy, home care, device replacement

## Abstract

**Background:**

Children with medical complexities needing palliative care often rely on tracheostomy and gastrostomy tubes. As home-based care gains priority to improve well-being and reduce hospitalizations, this study evaluates the safety and feasibility of home replacement of these devices.

**Methods:**

The study was a cross-sectional observational study of pediatric tracheostomy and/or gastrostomy patients followed at home by the Friuli-Venezia Giulia Regional Pediatric Palliative Care Centre at the Institute for Maternal and Child Health IRCCS “Burlo Garofolo”, Trieste. Data were consecutively collected between March 2020 and October 2024 by reviewing patients' medical records. A general satisfaction survey was additionally sent to caregivers.

**Results:**

The study sample included 14 children with a median age of 11 years (range 2–18 years). Among them, 3 (21%) had a tracheostomy, 7 (50%) had a gastrostomy and 4 (29%) had both devices. From 2020 to the present, 77 tracheostomy tube changes and 82 gastrostomy button changes were performed. No complications occurred. Families expressed high satisfaction with home device replacements, with nearly all preferring them over hospital-based changes.

**Conclusion:**

This study shows the safety and feasibility of home tracheostomy and gastrostomy replacement, highlighting the strong preference of patients' families for these procedures. By ensuring a secure approach, they help preserve the quality of life for both patients and their families.

## 1 Introduction

The prevalence of children with medical complexity (CMC) has significantly increased in recent years, largely driven by remarkable technological and medical advancements (1, 2). These innovations have led to higher survival rates among premature infants, children born with congenital anomalies, and those managing chronic or life-limiting conditions (1, 3, 4). While these developments highlight progress in pediatric healthcare, they have also resulted in a growing cohort of children requiring highly specialized and intensive medical care. Many of these children depend on advanced medical technologies, such as tracheostomies for respiratory support, gastrostomies for nutritional needs, and central venous access, all of which require a high level of expertise and continuous training for caregivers and healthcare providers (5, 6).

The responsibility of coordinating medical appointments, administering medications, and managing complex medical equipment falls primarily on caregivers, often leading to significant stress and fatigue. In response to the unique and multifaceted needs of this vulnerable population, pediatric palliative care (PPC) has emerged as a crucial field. PPC is designed to provide holistic support, addressing not only the children's medical needs but also their emotional, social, and psychological well-being, along with that of their families (2). The primary goal of PPC is to enhance the quality of life for both the child and his family, offering comprehensive care that extends beyond mere symptom management. PPC can be delivered in various settings, including hospitals, specialized pediatric units, and increasingly, within the home environment. In recent years, there has been a notable shift toward home-based care, driven by family preferences, as this model is often more conducive to the child's well-being (7, 8). Home-based care offers several advantages, including reduced stress from frequent hospital visits, the ability to maintain a sense of normalcy, and the creation of a more peaceful and familiar environment for the child and his siblings (9–13). As the demand for PPC continues to grow, particularly in home-based settings, healthcare systems must adapt to provide the necessary resources, training, and support to ensure that these children and their families receive the highest standard of care (14, 15).

Existing literature supports the safety, effectiveness, and family preference for certain medical procedures performed at home. Notably, at the Regional PPC Centre of the Institute for Maternal and Child Health, IRCCS “Burlo Garofolo” in Trieste, Italy, recent studies have explored home-based transfusions and chemotherapy administration, demonstrating excellent outcomes (16, 17). However, regarding tracheostomy and gastrostomy replacement, even in adults, there is a lack of literature describing its safety and feasibility in home settings. Certain prerequisites must be met to ensure safe implementation, including patient-specific factors, the home environment, caregiver motivation, and adequate procedural training, all of which require prior assessment. In Friuli-Venezia Giulia, since the onset of the COVID-19 pandemic in 2020, the PPC Network has developed a protocol for performing tracheostomy and gastrostomy replacement at home, carried out by trained pediatrician or an Ear, Nose, and Throat specialist (ENT) specialist. The pediatrician underwent a training period with the ENT team, participating in at least a dozen of in-hospital replacements and obtaining a Pediatric Advanced Life Support certification.

This study aims to evaluate the safety and effectiveness of home-based tracheostomy and gastrostomy replacements, as well as its impact on the quality of life of children and their families.

## 2 Methods

This cross-sectional observational study was conducted at the Regional Centre for PPC of IRCCS “Burlo Garofolo” in Trieste, Italy. Data were collected consecutively between March 2020 and October 2024. The study enrolled patients whose tracheostomy and/or gastrostomy device had been replaced at home by the PPC center's personnel during this period. Patients were excluded if their device was not replaced at home or if their families declined to participate.

The PPC center's personnel received training from surgeons and ENT specialists for gastrostomy and tracheostomy replacement, respectively. The training consisted of two phases: first, a session was conducted using a mannequin; then, at least five replacements were performed under the supervision of specialists, until the PPC personnel gained enough confidence to carry out the replacements independently.

Tracheostomy replacement was routinely performed once a month, with earlier replacement if obstruction or device failure occurred. Gastrostomy replacement, on the other hand, was carried out every four to six months, unless an issue arose earlier (e.g., displacement, rupture, etc.). Essential requirements during the procedures included: a suction pump, an oximeter, and at least two tracheostomy tubes (one of a smaller diameter in case of difficulties during insertion) for tracheostomy replacement; and a gastrostomy tube, gauzes, lubricating gel, and narrower catheters for insertion in case of complications during gastrostomy replacement. During home replacements caregivers were thought how to it by themselves, particularly in case of accidental displacement. A checklist was used to assess caregivers' readiness, then they could perform the following replacement by themselves at home.

The primary objective of the study was to assess the safety of performing device replacements at home, while the secondary objective focused on evaluating family satisfaction through a survey.

Adverse events associated with gastrostomy replacement included buried bumper syndrome, tube displacement, and bleeding. For tracheostomy replacement, adverse events considered were accidental decannulation, tube obstruction, and tracheal trauma. Caregivers can contact the PPC Service in case of any issue. The team remained at home for 30 min after each replacement in order to be ready to manage possible issues.

The family satisfaction component explored the impact of at-home medical device replacement on the perceived quality of life and stress levels of both the patients and their family. A five-point Likert-type scale, ranging from “not at all satisfied/fully disagree” to “very satisfied/strongly agree,” was used to evaluate parental satisfaction across several dimensions, supplemented by brief open-ended questions. The questions explored the level of stress perceived by both parents and children in the hospital and at home, the main sources of stress, the attitude of healthcare providers, and the occurrence of any complications. In addition, the questionnaire assessed potential savings in terms of transportation costs, time, and caregiver burden in the case of home-based replacements. In addition, the questionnaire assessed potential savings in terms of transportation costs, time, and caregiver burden in the case of home-based replacements. Moreover, through the questionnaire, data on nationality, main caregiver, caregivers' job, and the presence and age of other children were investigated. The complete satisfaction survey is available in the Supplementary Materials (Supplementary Material S1). Eligible families were invited via email, which included a detailed description of the study and a link to the survey.

Data were anonymously collected in a database, including the registration of each replacement and the schedule of the next one, in order to minimize potential reporting bias.

The Hospital Review Board approved the survey (ethics committee approval number 34/18), and parents had provided consent for the scientific use of medical records at the time of their child's initial admission.

## 3 Results

Fourteen patients were enrolled in the study, ten males and four females, with a median age at diagnosis of 11 years (range: 2–18 years, IQR: 7 years). Seven patients were tracheostomy-carrier, with a mean duration of 6.4 years and a median duration of three years; seven were PEG carriers (with a mean duration of seven years and a median of six years), and four had both medical devices. Three tracheostomized patients were also receiving continuous mechanical ventilation. The patients presented with diverse underlying conditions, which were categorized into three groups: genetic, neuromuscular, and neurological pathologies. Genetic disorders were the most common with six patients (42.86%), followed by neuromuscular disorders with five patients (35.71%) and neurological disorders with three patients (21.43%). Of these, two patients (14.29%) presented with spinal muscular atrophy type 1 (SMA1), two patients (14.29%) presented with *MECP2* duplication syndrome, two (14.29%) with hypoxic-ischemic encephalopathy, one (7.14%) with Aicardi Goutières syndrome, one (7.14%) with *MORC2* mutation syndrome, one (7.14%) with spastic tetraparesis secondary to cerebral hemorrhage (AVM rupture), one (7.14%) with Steinert myotonic dystrophy, one patient (7.14%) with autosomal recessive polycystic kidney, one (7.14%) with X-linked dominant mutation myopathy of the *FHL1* gene and lastly, and two patients (14.29%) with as yet unknown syndromes. The majority of patients with either tracheostomy or gastrostomy had the devices first positioned in the first three years of life. For those with only tracheostomy or gastrostomy, the device was first positioned at 0–3, 4–7 or 8–12 years in the same rate of the considered patients. Eleven (78.57%) patients were Italian, while three (21.43%) were of foreign nationality. For all patients, mothers were the main caregiver, without any employment in ten (71.43%) cases. Four (28.57%) families had no other children, while the remaining ten had additional offspring, the half of whom were younger than the patient receiving care. Patients' characteristics are summarized in Table 1.

**Table 1:** Patients' characteristics.

	Tracheostomy	Gastrostomy	Both
N patients (*N* = 14)	3 (21.43%)	7 (50%)	4 (28.57%)
Sex			
Female	1 (33.3%)	1 (14.29%)	2 (50%)
Male	2 (66.67%)	6 (85.71%)	2 (50%)
Median age (yrs) (IQR)	8 (14)	13 (6)	10.5 (11.5)
Disease			
Genetic syndrome	2 (66.67%)	3 (42.86%)	1 (25%)
Neuromuscular disease	1 (33.3%)	1 (14.28%)	3 (75%)
Neurological disease	0 (0%)	3 (42.86%)	0 (0%)
Nationality			
Italian	3 (100%)	5 (71.43%)	3 (75%)
Other	0 (0%)	2 (28.57%)	1 (25%)
Main caregiver			
Mother	3 (100%)	6 (85.71%)	4 (100%)
Father	0 (0%)	1 (14.29%)	0 (0%)
Mothers' employment status			
Without employment	2 (66.67%)	6 (85.71%)	2 (50%)
With employment	1 (33.3%)	1 (14.29%)	2 (50%)
Fathers' employment status			
Without employment	0 (0%)	1 (14.29%)	0 (0%)
With employment	3 (100%)	6 (85.71%)	4 (100%)
Number of family members			
3	1 (33.33%)	1 (14.29%)	2 (50%)
4	1 (33.33%)	2 (28.57%)	0 (0%)
5	1 (33.33%)	2 (28.57%)	2 (50%)
9	0 (0%)	2 (28.57%)	0 (0%)
Presence of other children			
No	1 (33.3%)	1 (14.29%)	2 (50%)
Yes	2 (66.67%)	6 (85.71%)	2 (50%)
Other children			
Younger	0 (0%)	2 (33.33%)	1 (50%)
Older	2 (100%)	2 (33.33%)	1 (50%)
Both	0 (0%)	2 (33.33%)	0 (0%)
Age of the child at device positioning			
0–3 years	1 (33.33%)	2 (28.57%)	3 (75%)
4–7 years	1 (33.33%)	2 (28.57%)	0 (0%)
8–12 years	1 (33.33%)	2 (28.57%)	0 (0%)
>12 years	0 (0%)	1 (14.29%)	1 (25%)

Regarding transportation and time issues in relation to hospital versus home replacement, most families required about one to two hours to bring the child to the hospital for the replacement, typically with a single caregiver, except in the case of children with both gastrostomy and tracheostomy. Most families used their own vehicle for transportation. Data on travel from home to hospital are summarized in Table 2.

**Table 2:** Means of transport and time issues.

	Tracheostomy	Gastrostomy	Both
Travel time (considering preparation time for the child and travel to the facility)			
<1 h	1 (33.3%)	0 (0%)	1 (25%)
1–2 h	1 (33.3%)	6 (85.7%)	2 (50%)
>2 h	1 (33.3%)	1 (14.3%)	1 (25%)
Median travel time (hour) (IQR)	1 (1.5)	2 (1.0)	1.5 (1.4)
More than one caregiver needed for the transport			
Yes	1 (33.3%)	2 (28.6%)	3 (75%)
No	2 (66.7%)	5 (71.4%)	1 (25%)
Means of transport used during therapy			
Personal car	3 (100%)	7 (100%)	2 (50%)
Private vehicle	0 (0%)	0 (0%)	2 (50%)

All families responded to the anonymous survey, a few after a reminder. Data are reported by dividing the patients into three groups: those with only a tracheostomy (n. 3), those with only a gastrostomy (n. 7), and those with both devices (n. 4).

### 3.1 Safety and adverse events

Over the study period, a total of 77 tracheostomy and 82 gastrostomy home changes were performed, and no adverse events were reported. The median time needed to move the child from home to the hospital, considering preparation time for the child and travel to the facility, was reported to be 1 h for children with tracheostomy, two hours for children with gastrostomy and one hour and 30 min for children with both devices. For children with tracheostomy, in one out of three cases (33.33%), parents reported that more than one caregiver is needed to move the child to the hospital. Instead, for those with gastrostomy, in two out of seven cases (28.57%), parents required more than one caregiver to be moved. Finally, for children with both devices, in three out of four cases (75.00%), parents required more than one caregiver. Regarding transportation from home to the hospital, many children needed to be transported by ambulance or with the help of volunteer associations. Among families with children with a tracheostomy, none used a private vehicle to transport their child to the hospital. Similarly, among those with a gastrostomy, no one used a private vehicle. However, among children with both devices, two out of four (50.00%) use a private vehicle for transportation.

### 3.2 Child stress

**Figure 1:**
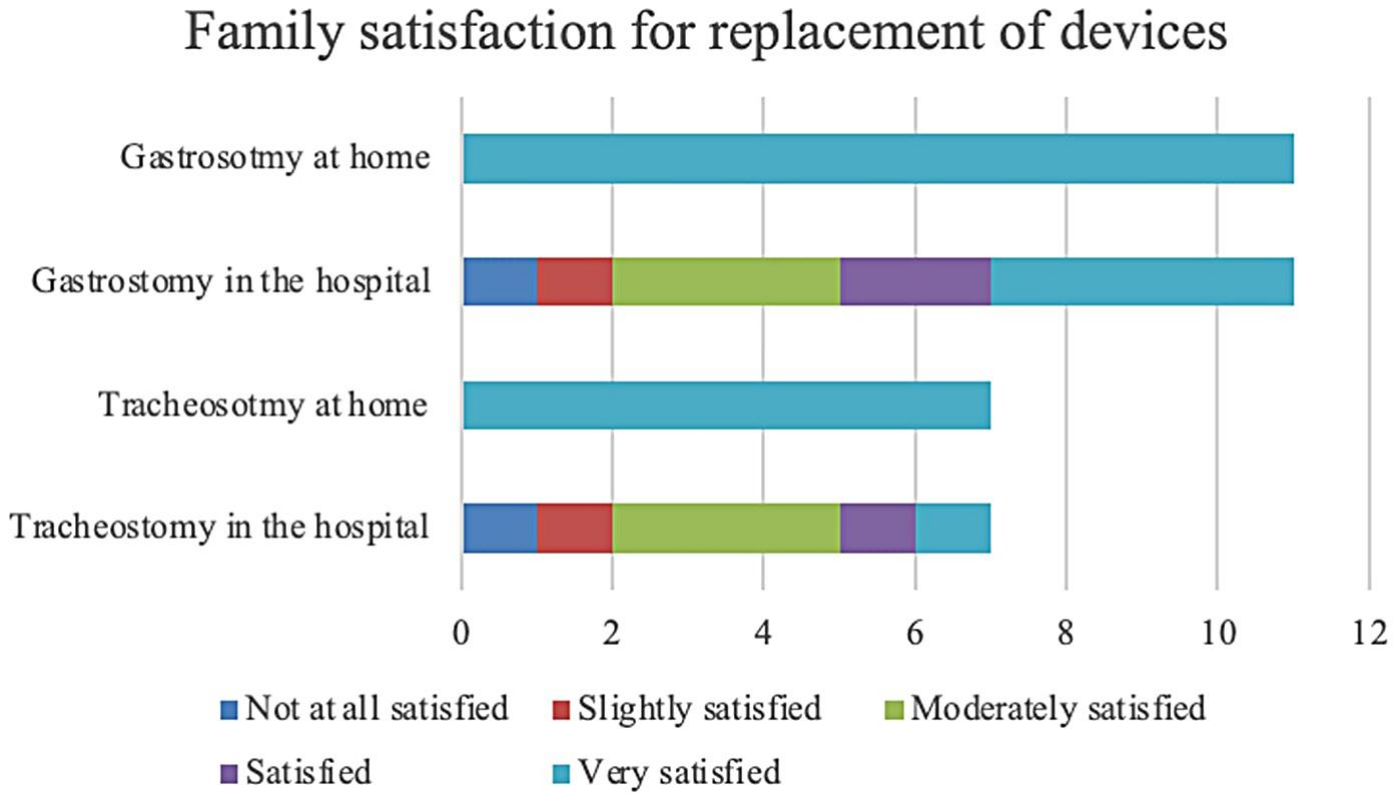
Child stress for replacement of devices.

Regarding the child's perceived stress level during tracheostomy replacement in the hospital, the average Likert scale rating reported by families was 3.86/5. Similarly, a mean score of 4/5 was reported for gastrostomy replacement in the hospital. When the procedure was performed at home, the child's perceived stress level for tracheostomy replacement was consistently rated at an average of 1.29/5. Likewise, gastrostomy replacement at home had a mean rating of 1.27/5. Child's perceived stress levels are showed in Figure 1.

### 3.3 Family satisfaction

**Figure 2:**
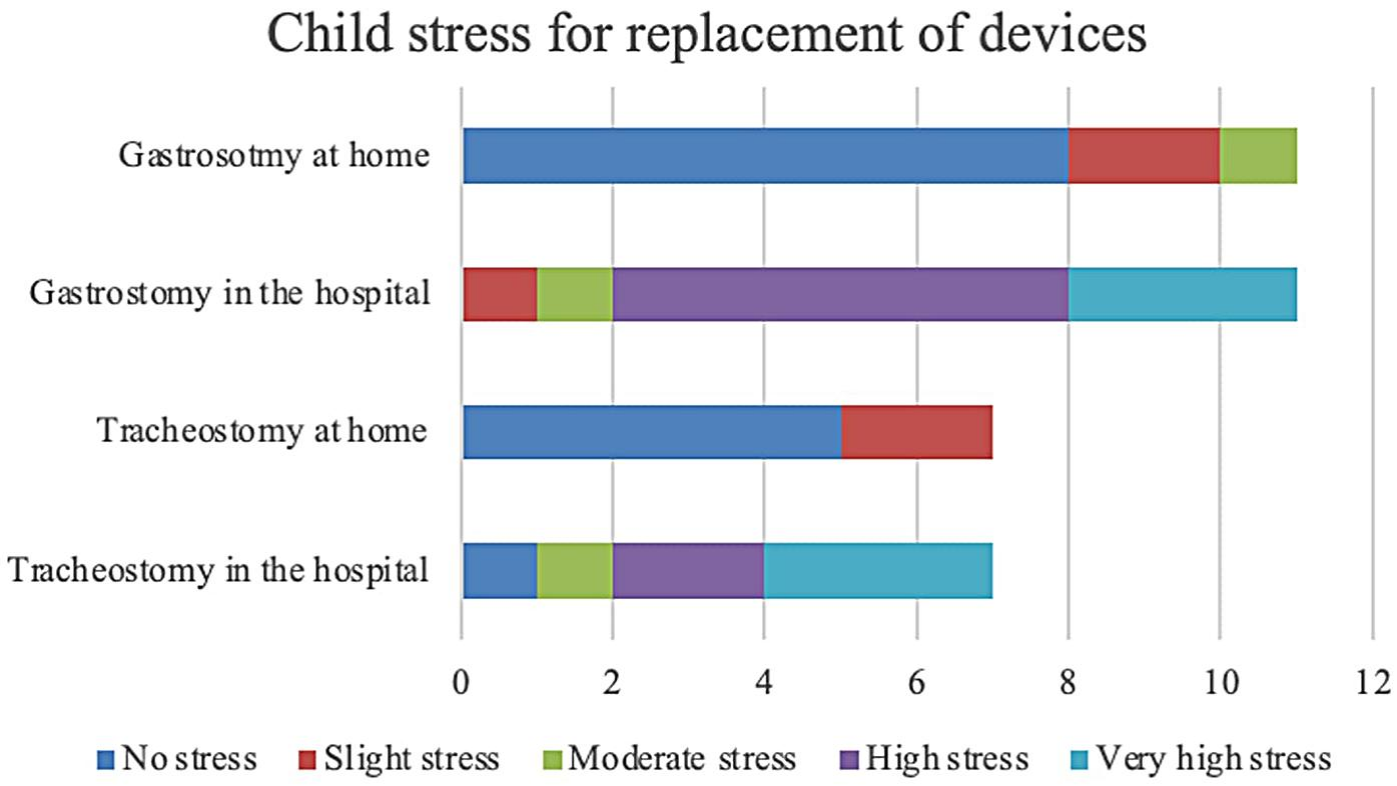
Family satisfaction for replacement of devices.

The assessment of family satisfaction with home tracheostomy cannula replacement showed a 100% satisfaction rate. Moreover, when asked about their preferred location for the procedure, 85.7% of families indicated a preference for home. Similarly, family satisfaction with home gastrostomy button replacement was evaluated, with 90.9% of families rating their satisfaction as “strongly agree” (5/5) and 9.1% as “agree” (4/5). Furthermore, when asked about their preferred setting for the procedure, all families (100%) expressed a preference for home. Family's satisfaction is showed in Figure 2.

## 4 Discussion

This study highlights the safety of home-based tracheostomy and gastrostomy replacements in a cohort of CMC followed by a PPC team. CMC require specialized care and ongoing medical interventions due to the significant technical and technological complexities involved in their treatment. The prevalence of both gastrostomies and tracheostomies in this population has risen significantly in recent years, placing considerable burdens on families. Indeed, parents of CMC face substantial caregiving challenges, emotionally, financially and technically (11). Previous literature has suggested that home-based care in the pediatric palliative care setting is preferable, as it allows the child and their family to remain in a familiar environment and maintain a routine closer to normal life (7, 8, 18). Our study reinforces this finding. We observed that the level of perceived family stress associated with home-based replacements was significantly lower compared to hospital-based replacements. Furthermore, family satisfaction was extremely high, with nearly all families opting for device replacements at home. This preference was primarily driven by the time and cost savings experienced by families. Although our study did not extensively investigate these savings, it provides a foundation for future research. Time savings were reflected in parents needing fewer vacation days and siblings missing fewer school days. In terms of cost savings, families benefited from not needing to travel to the hospital, particularly when relying on private transportation.

The limitations of our study include the retrospective nature, the relatively small sample size, and the use of non-standardized, non-validated questionnaires. However, the study's strengths lie in its family-centered approach, assessing perceived stress levels and satisfaction with home vs. hospital-based device replacements. Moreover, we collected demographic data, but no specific statistical analysis was performed e.g., in relation to nationality, working caregiver or the presence of other siblings, even though we did not report any significant complication for all patients. Additionally, we did not conduct a detailed time and cost analysis, as multiple variables—such as different healthcare professionals involved and the simultaneous execution of other procedures during scheduled visits—would have complicated the interpretation of results. Notably, this study appears to be the first global report on the safety, effectiveness, and family satisfaction of home-based tracheostomy and gastrostomy replacements in pediatric patients. As the number of children with medical complexities continues to grow, the need for ongoing support, specialized caregiver training, and comprehensive care strategies becomes increasingly critical. In particular, continuous efforts are needed to enhance caregiver education, which plays a key role in fostering greater autonomy and confidence in managing home care devices. Moreover, training other healthcare professionals within the family's local area is essential to strengthening expertise and collaboration within the palliative care network. Empowering families remains a primary long-term objective, as it enhances both patient and caregiver well-being by promoting greater independence and security. Future studies should explore the financial and time-saving benefits of home-based procedures in greater depth. Expanding research to include a larger patient population across different regions—or even a nationwide analysis—could provide further insights into the broader advantages of this practice.

This study emphasizes that performing tracheostomy and gastrostomy replacements at home is both safe and feasible in the presence of a trained team and in case of favorable healthcare organization setting and selected families, offering significant benefits in preserving the quality of life for children and their families. This is a preliminary, hypothesis-generating report which may pave the way to future wider and multicenter studies.

## Data Availability

The original contributions presented in the study are included in the article/Supplementary Material, further inquiries can be directed to the corresponding author.
