# Supplementary material for: Safety and feasibility of tracheostomy and gastrostomy home replacement: a five-year experience from a palliative care center
Source: Front Pediatr. 2025 Sep 4;13:1644830. doi: 10.3389/fped.2025.1644830 (PMC12443695; doi:10.3389/fped.2025.1644830)
Supplement: Supplementary file 1 [file Table1.docx]

Supplementary Material

**Supplementary file 1: Satisfaction survey**

1. **Gastrostomy (PEG) replacement**

| **Patient’s sex** | Male | | | | | | | | | | Female | | | | | | | | | |
| --- | --- | --- | --- | --- | --- | --- | --- | --- | --- | --- | --- | --- | --- | --- | --- | --- | --- | --- | --- | --- |
| **Patient’s age (years)** |  | | | | | | | | | | | | | | | | | | | |
| **Nationality** |  | | | | | | | | | | | | | | | | | | | |
| **Type of disease** | Genetic | | | Neuromuscular | | | | | Neurological | | | | | Oncological | | | | | Other | |
| **Your job** |  | | | | | | | | | | | | | | | | | | | |
| **Other caregiver’s job** |  | | | | | | | | | | | | | | | | | | | |
| **Other children** | Yes | | | | | | | | | | | No | | | | | | | | |
|  | If yes, age: | | | | | | | | | | | | | | | | | | | |
| **Main caregiver** | Mother | | | | | | Father | | | | | | | | Other | | | | | |
| **PEG home management** | | | | | | | | | | | | | | | | | | | | |
| **For how many years has your child had PEG?** | | | | | | | | | |  | | | | | | | | | | |
| **How much time do you need daily for the PEG management?** | | | | | | | | | |  | | | | | | | | | | |
| **How much time do you need to come to the hospital, considering the time needed to prepare your child and to travel?** | | | | | | | | | |  | | | | | | | | | | |
| **Is more than one person needed to bring your child to the hospital?** | | | | | | | | | | Yes | | | | | | | | No | | |
| **Is a special means of transport needed (es. ambulance) to bring the child to the hospital?** | | | | | | | | | | Yes | | | | | | | | No | | |
| **In hospital PEG replacement** | | | | | | | | | | | | | | | | | | | | |
| **How stressful is the PEG replacement in hospital for you?** | Zero | 1 | | | 2 | | | | 3 | | | | 4 | | | | | 5 | Very much | |
| **How stressful is the PEG replacement in hospital for the child?** | Zero | 1 | | | 2 | | | | 3 | | | | 4 | | | | | 5 | Very much | |
| **If you consider the procedure in hospital as stressful for the child, can you tell the reasons?** | | | | | | | | |  | | | | | | | | | | | |
| **During the PEG replacement in hospital, have complications ever occurred? If yes, which one?** | | | | | | | | |  | | | | | | | | | | | |
| **Which is your level of satisfaction about the PEG replacement in hospital?** | Not at all | 1 | | | 2 | | | | 3 | | | | 4 | | | 5 | | | Very satisfied | |
| **How was the healthcare provider’s attitude in hospital?** | | | | | | | | | | | | | | | | | | | | |
| **Welcoming** | Not at all | 1 | | | 2 | | | | 3 | | | | 4 | | | 5 | | | Very much | |
| **Kind** | Not at all | 1 | | | 2 | | | | 3 | | | | 4 | | | 5 | | | Very much | |
| **Able to clarify doubts** | Not at all | 1 | | | 2 | | | | 3 | | | | 4 | | | 5 | | | Very much | |
| **Available to listen (time devoted)** | Not at all | 1 | | | 2 | | | | 3 | | | | 4 | | | 5 | | | Very much | |
| **PEG replacement at home** | | | | | | | | | | | | | | | | | | | | |
| **How many times have PEG been replaced at home?** | | | | | | | | |  | | | | | | | | | | | |
| **Who suggested replacing the PEG at home?** | | | | | | | | |  | | | | | | | | | | | |
| **Which healthcare providers replaced the PEG at home?** | | | | | | | | |  | | | | | | | | | | | |
| **Have you ever replaced the PEG at home by yourself?** | | | | | | | | |  | | | | | | | | | | | |
| **Who taught you how to replace the PEG?** | | | | | | | | |  | | | | | | | | | | | |
| **Do you feel confident in replacing the PEG?** | | | | | | | | |  | | | | | | | | | | | |
| **How stressful is the PEG replacement at home for you?** | Not at all | 1 | | | 2 | | | | 3 | | | | 4 | | | | | 5 | | Very much |
| **How stressful is the PEG replacement at home for the child?** | Not at all | 1 | | | 2 | | | | 3 | | | | 4 | | | | | 5 | | Very much |
| **If you consider the procedure at home as stressful for the child, can you tell the reasons?** | | | | | | | | |  | | | | | | | | | | | |
| **During the PEG replacement at home, have complications ever occurred? If yes, which one?** | | | | | | | | |  | | | | | | | | | | | |
| **Which is your level of satisfaction about the PEG replacement at home?** | Not at all | | 1 | | | 2 | | | 3 | | | | 4 | | | | | 5 | | Very satisfied |
| **How was the healthcare provider’s attitude at home?** | | | | | | | | | | | | | | | | | | | | |
| **Welcoming** | Not at all | | 1 | | | 2 | | | 3 | | | | 4 | | | | | 5 | | Very much |
| **Kind** | Not at all | | 1 | | | 2 | | | 3 | | | | 4 | | | | | 5 | | Very much |
| **Clarifier of doubts** | Not at all | | 1 | | | 2 | | | 3 | | | | 4 | | | | | 5 | | Very much |
| **Available to listen (time devoted)** | Not at all | | 1 | | | 2 | | | 3 | | | | 4 | | | | | 5 | | Very much |
| **Comparison between PEG replacement in hospital and at home** | | | | | | | | | | | | | | | | | | | | |
| **How much do you agree with the following statements?** | | | | | | | | | | | | | | | | | | | | |
| **PEG replacement at home reduces the cost of transport** | Fully disagree | | 1 | | | 2 | | | 3 | | | | 4 | | | | | 5 | | Strongly agree |
| **PEG replacement at home helps save time** | Fully disagree | | 1 | | | 2 | | | 3 | | | | 4 | | | | | 5 | | Strongly agree |
| **PEG replacement at home reduces the burden on caregivers** | Fully disagree | | 1 | | | 2 | | | 3 | | | | 4 | | | | | 5 | | Strongly agree |
| **PEG replacement at home is as safe as the one in hospital** | Fully disagree | | 1 | | | 2 | | | 3 | | | | 4 | | | | | 5 | | Strongly agree |
| **The quality of assistance during PEG replacement at home is equal to that in hospital** | Fully disagree | | 1 | | | 2 | | | 3 | | | | 4 | | | | | 5 | | Strongly agree |
| **Overall, where do you prefer to have your child’s PEG replaced?** | | | | | | | | In hospital | | | | | | | | | At home | | | |

1. **Regarding tracheostomy replacement**

| **Patient’s sex** | Male | | | | | | | | | | Female | | | | | | | | | | | |
| --- | --- | --- | --- | --- | --- | --- | --- | --- | --- | --- | --- | --- | --- | --- | --- | --- | --- | --- | --- | --- | --- | --- |
| **Patient’s age (years)** |  | | | | | | | | | | | | | | | | | | | | | |
| **Nationality** |  | | | | | | | | | | | | | | | | | | | | | |
| **Type of disease** | Genetic | | | Neuromuscular | | | | | Neurological | | | | | | Oncological | | | | | | Other | |
| **Your job** |  | | | | | | | | | | | | | | | | | | | | | |
| **Other caregiver’s job** |  | | | | | | | | | | | | | | | | | | | | | |
| **Other children** | Yes | | | | | | | | | | | No | | | | | | | | | | |
|  | If yes, age: | | | | | | | | | | | | | | | | | | | | | |
| **Main caregiver** | Mother | | | | | | Father | | | | | | | | | Other | | | | | | |
| **Tracheostomy home management** | | | | | | | | | | | | | | | | | | | | | | |
| **For how many years has your child had tracheostomy?** | | | | | | | | | | | | |  | | | | | | | | | |
| **How much time do you need daily for the tracheostomy management?** | | | | | | | | | | | | |  | | | | | | | | | |
| **How much time do you need to come to the hospital, considering the time needed to prepare your child and to travel?** | | | | | | | | | | | | |  | | | | | | | | | |
| **Is more than one person needed to bring your child to the hospital?** | | | | | | | | | | | | | Yes | | | | | | | No | | |
| **Is a special mean of transport needed (es. ambulance) to bring the child to the hospital?** | | | | | | | | | | | | | Yes | | | | | | | No | | |
| **In hospital tracheostomy replacement** | | | | | | | | | | | | | | | | | | | | | | |
| **How stressful is the tracheostomy replacement in hospital for you?** | Not at all | 1 | | | 2 | | | | 3 | | | | | 4 | | | | | 5 | | Very much | |
| **How stressful is the tracheostomy replacement in hospital for the child?** | Not at all | 1 | | | 2 | | | | 3 | | | | | 4 | | | | | 5 | | Very much | |
| **If you consider the procedure in hospital as stressful for the child, can you tell the reasons?** | | | | | | | | |  | | | | | | | | | | | | | |
| **During the tracheostomy replacement in hospital, have complications ever occurred? If yes, which one?** | | | | | | | | |  | | | | | | | | | | | | | |
| **Which is your level of satisfaction about the tracheostomy replacement in hospital?** | Not at all | 1 | | | 2 | | | | 3 | | | | | 4 | | | 5 | | | | Very much | |
| **How was the healthcare provider’s attitude in hospital?** | | | | | | | | | | | | | | | | | | | | | | |
| **Welcoming** | Not at all | 1 | | | 2 | | | | 3 | | | | | 4 | | | 5 | | | | Very much | |
| **Kind** | Not at all | 1 | | | 2 | | | | 3 | | | | | 4 | | | 5 | | | | Very much | |
| **Clarifier of doubts** | Not at all | 1 | | | 2 | | | | 3 | | | | | 4 | | | 5 | | | | Very much | |
| **Available to listen (time devoted)** | Not at all | 1 | | | 2 | | | | 3 | | | | | 4 | | | 5 | | | | Very much | |
| **Tracheostomy replacement at home** | | | | | | | | | | | | | | | | | | | | | | |
| **How many times have tracheostomy been replaced at home?** | | | | | | | | | |  | | | | | | | | | | | | |
| **Who suggested replacing the tracheostomy at home?** | | | | | | | | | |  | | | | | | | | | | | | |
| **Which healthcare providers replaced the tracheostomy at home?** | | | | | | | | | |  | | | | | | | | | | | | |
| **Have you ever replaced the tracheostomy at home by yourself?** | | | | | | | | | |  | | | | | | | | | | | | |
| **Who taught you how to replace the tracheostomy?** | | | | | | | | | |  | | | | | | | | | | | | |
| **Do you feel confident in replacing the tracheostomy?** | | | | | | | | | |  | | | | | | | | | | | | |
| **How stressful is the tracheostomy replacement at home for you?** | Not at all | 1 | | | 2 | | | | 3 | | | | | 4 | | | | | 5 | | | Very much |
| **How stressful is the tracheostomy replacement at home for the child?** | Not at all | 1 | | | 2 | | | | 3 | | | | | 4 | | | | | 5 | | | Very much |
| **If you consider the procedure at home as stressful for the child, can you tell the reasons?** | | | | | | | | |  | | | | | | | | | | | | | |
| **During the tracheostomy replacement at home, have complications ever occurred? If yes, which one?** | | | | | | | | |  | | | | | | | | | | | | | |
| **Which is your level of satisfaction about the tracheostomy replacement at home?** | Not at all | | 1 | | | 2 | | | 3 | | | | | 4 | | | | | 5 | | | Very much |
| **How was the healthcare provider’s attitude at home?** | | | | | | | | | | | | | | | | | | | | | | |
| **Welcoming** | Not at all | | 1 | | | 2 | | | 3 | | | | | 4 | | | | | 5 | | | Very much |
| **Kind** | Not at all | | 1 | | | 2 | | | 3 | | | | | 4 | | | | | 5 | | | Very much |
| **Clarifier of doubts** | Not at all | | 1 | | | 2 | | | 3 | | | | | 4 | | | | | 5 | | | Very much |
| **Available to listen (time devoted)** | Not at all | | 1 | | | 2 | | | 3 | | | | | 4 | | | | | 5 | | | Very much |
| **Comparison between PEG replacement in hospital and at home** | | | | | | | | | | | | | | | | | | | | | | |
| **How much do you agree with the following statements?** | | | | | | | | | | | | | | | | | | | | | | |
| **Tracheostomy replacement at home reduces the cost of transport** | Fully disagree | | 1 | | | 2 | | | 3 | | | | | 4 | | | | | 5 | | | Strongly agree |
| **Tracheostomy replacement at home helps save time** | Fully disagree | | 1 | | | 2 | | | 3 | | | | | 4 | | | | | 5 | | | Strongly agree |
| **Tracheostomy replacement at home reduces the burden on caregivers** | Fully disagree | | 1 | | | 2 | | | 3 | | | | | 4 | | | | | 5 | | | Strongly agree |
| **Tracheostomy replacement at home is as safe as the one in hospital** | Fully disagree | | 1 | | | 2 | | | 3 | | | | | 4 | | | | | 5 | | | Strongly agree |
| **The quality of assistance during tracheostomy replacement at home is equal to that in hospital** | Fully disagree | | 1 | | | 2 | | | 3 | | | | | 4 | | | | | 5 | | | Strongly agree |
| **Overall, where do you prefer to have your child’s tracheostomy replaced?** | | | | | | | | In hospital | | | | | | | | | | At home | | | | |
